# Supplementary material for: Predicting prostate cancer progression: protocol for a retrospective cohort study to identify prognostic factors for prostate cancer outcomes using routine primary care data
Source: BMJ Open. 2018 Jan 31;8(1):e019409. doi: 10.1136/bmjopen-2017-019409 (PMC5829815; doi:10.1136/bmjopen-2017-019409)
Supplement: Author's manuscript [file bmjopen-2017-019409.draft_revisions.pdf]

BMJ Open

BMJ Open is committed to open peer review. As part of this commitment we make the peer review history of every article we publish publicly available.

When an article is published we post the peer reviewers' comments and the authors' responses online. We also post the versions of the paper that were used during peer review. These are the versions that the peer review comments apply to.

The versions of the paper that follow are the versions that were submitted during the peer review process. They are not the versions of record or the final published versions. They should not be cited or distributed as the published version of this manuscript.

BMJ Open is an open access journal and the full, final, typeset and author-corrected version of record of the manuscript is available on our site with no access controls, subscription charges or pay-per-view fees (http://bmjopen.bmj.com).

If you have any questions on BMJ Open's open peer review process please email editorial.bmjopen@bmj.com

BMJ Open

Predicting prostate cancer progression: Retrospective cohort study to identify prognostic factors for prostate cancer outcomes using routine primary care data

Journal:	*BMJ Open*
Manuscript ID	bmjopen-2017-019409
Article Type:	Protocol
Date Submitted by the Author:	31-Aug-2017
Complete List of Authors:	Merriel, Samuel; Univ Bristol, Centre for Academic Primary Care<br>Martin, Richard; University of Bristol, Social Medicine<br>May, Margaret; University of Bristol School of Social and Community Medicine,
Keywords:	Epidemiology < ONCOLOGY, Urological tumours < ONCOLOGY, Prostate disease < UROLOGY

SCHOLARONE™
Manuscripts

For peer review only

'Predicting prostate cancer progression: Retrospective cohort study to identify prognostic factors for prostate cancer outcomes using routine primary care data'

Dr Samuel W D Merriel[1], Prof Margaret T May[2], Prof Richard M Martin[2]

1 Centre for Academic Primary Care, University of Bristol, UK

2 School of Social & Community Medicine, University of Bristol, UK

Corresponding author

Dr Samuel William David Merriel

Centre for Academic Primary Care

School of Social & Community Medicine

University of Bristol

39 Whatley Road

Clifton, Bristol, UK

Sam.merriel@bristol.ac.uk

0117 928 9000

Keywords – prostate cancer, prognosis, primary care

Word count – 1590

For peer review only

Abstract

Introduction

Prostate cancer is the most common cancer in UK males, with nearly 40,000 men diagnosed in 2014, and the second commonest cause of male cancer-related mortality. The clinical conundrum is that most men live with prostate cancer rather than die from it, while existing treatments have significant associated morbidity. Recent studies have shown very low mortality rates (1% after a median 10-years follow-up), and no treatment related reductions in mortality, in men with localised prostate cancer. This study will identify prognostic factors associated with prostate cancer progression to help differentiate aggressive from more indolent tumours in men with localised disease at diagnosis, and so inform the decision to adopt conservative (active surveillance) or radical (surgery or radiotherapy) management strategies.

Methods & analysis

The Clinical Practice Research Datalink (CPRD) contains 57,318 men who were diagnosed with prostate cancer between 01/01/1987 and 31/12/2016. These men will be linked to the Office for National Statistics (ONS) and the National Cancer Registration and Analysis Service (NCRAS) registry databases for mortality, TNM stage, Gleason grade, and treatment data. Men with a diagnosis date prior to 01/01/1987 and men with lymph node or distant metastases at diagnosis will be excluded. A priori determined prognostic factors potentially associated with prostate cancer mortality, the end point of cancer progression, will be measured at baseline, and the participants followed through to development of cancer progression, death, or the end of the follow-up period (31/12/2016). Cox proportional hazards regression will be used to estimate crude and mutually adjusted hazard ratios. Mortality risk will be predicted using flexible parametric survival models that can accurately fit the shape of the hazard function.

Ethics & dissemination

This study protocol has approval from the Independent Scientific Advisory Committee (ISAC) for the UK Medicines and Healthcare products Regulatory Agency (MHRA) Database

For peer review only

Research (protocol 17_041). The findings will be presented in peer reviewed journals and local CPRD researcher meetings.

For peer review only

Strengths and limitations

- The study cohort is drawn from the CPRD, a large, representative UK primary care dataset, with linked cancer registry and ONS data
- Predicting cancer progression is a more clinically useful outcome than simply detecting localised disease in patients with a disease that is often indolent and slow-growing
- Flexible parametric modelling will be used to control for the effects of intermediate variables and prognostic factor combinations will be used
- Cancer registry data and ONS mortality data are not available for the full study period, and CPRD data may not be complete

For peer review only

Introduction

Prostate cancer is the most commonly diagnosed cancer in males, and the second most commonly diagnosed cancer in the United Kingdom (UK). 39,741 new cases of prostate cancer were diagnosed in England in 2014, with an age-standardised incidence rate of 177.8 per 100,000 males(1). In the same year there were approximately 11,300 prostate cancer deaths in the UK, making prostate cancer the second most common cause of cancer death in males. Nevertheless, survival rates for prostate cancer are relatively high compared to other cancer types. The overall 5-year age-standardised net survival for men with prostate cancer in England was 83.6%, and the predicted 10-year survival for men in England diagnosed with prostate cancer in 2015 is 79.9%(2). This suggests many men diagnosed with prostate cancer have indolent disease. A key clinical conundrum relates to distinguishing men with slow-growing tumours that could be managed conservatively with active monitoring from more aggressive, potentially fatal disease that may require more radical intervention.

Prostate cancer can be detected in men in different ways. GPs need to consider the possibility of prostate cancer in men presenting with lower urinary tract symptoms, erectile dysfunction or visible haematuria. Asymptomatic men may also be found to have raised prostate specific antigen (PSA) levels, and need to be referred for further investigation(3). However, the use of PSA as a screening and prognostic biomarker remains controversial(4,5), and GPs(6-9), and patients(10) have mixed views about its utility in informing investigation and treatment decisions for prostate cancer. Other screening methods for predicting prostate cancer severity have been tested, such as the STHLM-3 model(11), which did not look at risk of progression and relies on genetic biomarkers that are not readily available in primary care at this time.

The Bristol-based *ProtecT* multi-centre trial randomised men with clinically localised prostate cancer to either active monitoring, radical surgery (prostatectomy) or radical radiotherapy. After a median of 10 years' follow-up there was no difference in prostate cancer mortality. Overall, the 10-year mortality rates were very low (1%), and men randomised to active monitoring were at an increased risk of clinical progression and development of metastatic disease (22.9 per 1,000 person years follow-up) compared to the

For peer review only

radical treatment arms (8.9 and 9 per 1,000 person years respectively).(12) Men receiving surgery or radiotherapy reported more adverse effects on urinary, sexual and bowel function compared to the active monitoring cohort.(13) This was broadly consistent with other studies of prostate cancer treatment.(14-18) Identifying factors associated with prostate cancer progression may help determine the risk for men having more aggressive prostate cancer, and inform shared decision making about whether to undergo radical treatments or choose active monitoring.

Cancer progression is well defined is cancer treatment trials, following the widely used RECIST criteria.(19) However, the concept of cancer progression in prognostic studies is much less well defined or consistently applied. The *ProtecT* trial defined prostate cancer progression as the occurrence of any of the following events: evidence of metastasis, development of T3/T4 disease, commencing long-term androgen therapy, ureteric obstruction, rectal fistula, and new need for catheter.(12) Several prognostic factors have been identified that may be associated with prostate cancer mortality, the endpoint of prostate cancer progression. These include demographic(20), genetic(21), physiological(22,23), co-morbidity(24-27), lifestyle(28-33), biochemical(34,35) and medication(36,37) factors. The strength of evidence for these prognostics factors varies, and for many others it is conflicting(38-44).

Primary care medical records contain a wealth of information on a patient's medical history, medications, family history, and investigation results(45). The Clinical Practice Research Datalink (CPRD)(46) is a large UK primary care research database representative of the general population, with links to many other relevant healthcare data registries and Office for National Statistics (ONS) data. This information is already used for many risk prediction tools in primary care settings to predict outcomes and inform treatment decisions. Examples include QCancer(47), which predicts a patient's absolute risk of future cancer diagnosis. To date there are no risk prediction tools for cancer progression used in clinical practice.

This study aims to establish which risk factors are associated with prostate cancer progression using primary care medical records data. These findings, in combination with

For peer review only

metabolomic and genomic data, will inform the development of a clinical risk prediction model for the progression of prostate cancer following diagnosis.

For peer review only

Methods and analysis

Within the CPRD dataset, at least 57,318 men had a diagnosis of prostate cancer made between 01/01/1987 and 31/12/2016, of whom 22,080 have a recorded date of death. These men will form the basis of the study cohort. Additional mortality, staging, and treatment data will be obtained by using each man's NHS number to link them to the ONS (available from 01/01/1998) and National Cancer Registration and Analysis Service (NCRAS) (available from 01/01/1990) databases. The index date will be the date the diagnosis of prostate cancer was first entered into the primary care medical record. From this date, the men will be followed until the date of their death, the development of prostate cancer progression, or the end of the cohort period, whichever is later. Men with a diagnosis date prior to 01/01/1987, and men with lymph node or distant metastases at diagnosis, will be excluded from the analysis.

Each of the hypothesised prognostic factors for prostate cancer mortality identified a priori (see Table 1) will be recorded as an 'exposure' if it is entered into the patient record at the study baseline (index date), or recorded before the diagnosis of prostate cancer is entered. Continuous variables, such as height, weight and the biochemical markers, will be measured according to the most recent result prior to the coding of a diagnosis of prostate cancer within the study time period. Genetic factors, lifestyle exposures, medications and co-morbidities will be considered in a binary manner in relation to their presence or absence at the index date. Missing data will be controlled for using multiple imputation methods.(48)

**Category**	**Prognostic factor(s)**
Basic demographics	Age, date of birth, post code (to compute a measure of deprivation), ethnicity
Physiological	Height, weight, waist circumference, waist:hip ratio
Genetic	Family history of prostate cancer
Biochemical	Triglycerides, HDL cholesterol, LDL cholesterol, VLDL cholesterol, HbA1c, CRP, Ferritin, Haemoglobin, Albumin, Serum

For peer review only

	glucose, Plasma glucose, Lead
Lifestyle	Current smoker, Ex-smoker, In relationship, Alcohol use
Medications	Simvastatin, Atorvastatin, Metformin, Aspirin, Atenolol, Bisoprolol, Sotalol, Labetalol, Carvedilol, Nebivolol, Metoprolol, Propanolol, Finasteride, Dutasteride, Colecalciferol, Ergocalciferol, Alfacalcidol
Co-morbidities	Type 2 Diabetes Mellitus, Ischaemic heart disease, Stroke, Peripheral vascular disease, Benign prostatic hypertrophy, Chronic Obstructive Pulmonary Disease (COPD)

Table 1 – Prognostic factors to be assessed

HDL – High Density Lipoprotein; LDL – Low Density Lipoprotein; VLDL – Very Low Density Lipoprotein; HbA1c – Haemoglobin A1c; CRP – C-Reactive Protein

To achieve 95% power and detect a difference in hazard ratios of 0.5 in prostate cancer mortality for a binary risk factor using an alpha of 0.05, a sample of at least 8,762 men with prostate cancer would be required, assuming a 1% annual mortality rate over a median 10-year follow-up.

The primary outcome measure will be prostate cancer mortality, provided by linked cancer registry data. Secondary outcome measures of prostate cancer progression will include all-cause mortality, change from localised to metastatic disease, and commencing anti-androgen therapy or chemotherapy. We will use whether the treatment recorded in the registry is stated to be localised (i.e. 1 tumour treated) or systemic (i.e. >1 tumour treated) to help distinguish between early and advanced disease.

Descriptive statistics will be used to summarise the basic demographic details of the men. The prevalence of the pre-selected putative prognostic factors will be calculated and presented. Cox proportional hazards regression will be used to estimate the crude and

For peer review only

mutually adjusted hazard ratios (with 95% confidence intervals) for prostate cancer and all-cause mortality according to the prognostic factors. Related prognostic factors, such as smoking and COPD, will also be grouped to account for potential intermediate variables. This analysis will be repeated with stratification by stage at diagnosis. In order to allow for flexibility in the shape of the cumulative hazard function we will use flexible parametric survival models(49) for prognostic modelling. These models incorporate cubic spline terms in the log cumulative hazard function and are based on weibull, loglogistic or lognormal distributions of survival time. We will check for non-linearities in the effects of continuous predictors using fractional polynomials(50) and also test for time varying effects of prognostic factors. We will determine mortality risk in groups defined by important prognostic factors. To asses competing risks, we will use cause–specific survival analysis to estimate at 1, 2, 5 and 10 years post prostate cancer diagnosis the contribution of prostate cancer mortality to overall mortality in those who have died by prognostic factor combinations(51).

For peer review only

Ethics and dissemination

This study protocol has approval from the Independent Scientific Advisory Committee (ISAC) for the UK Medicines and Healthcare products Regulatory Agency (MHRA) Database Research (protocol 17_041).

The findings of this study will be submitted as a manuscript to peer reviewed journal to aid dissemination to clinicians and other researchers in the field. It will also be presented and discussed at local CPRD working groups to inform other researchers' methods using the CPRD database. Subsequent studies of the prediction tool, based on this piece of research, will involve clinicians at every stage to ensure the final tool is acceptable for use in clinical practice.

For peer review only

Conclusions

This study will lay the foundation for the development of a clinically useful risk prediction tool. Clinicians will be able to use the tool, inputting routine primary care data, to improve shared decision making about an individual's prognosis and, if validated and shown effective in trials, inform their practice when deciding with patients whether to undergo radical surgery or radiotherapy or be followed up conservatively using active monitoring.

Patients will also benefit from this work in other ways. They will be able to receive more information from GPs and NHS specialists about the risk of progression of their prostate cancer, and they will be able to decide within a shared decision-making framework with their doctors about the potential benefits and harms of undergoing radical treatment or active monitoring.

For peer review only

FUNDING

NIHR CLAHRC West funded the CPRD data costs for this study. SM is the recipient of an NIHR academic clinical fellowship.

DATA SHARING STATEMENT

There is no data to share from this protocol manuscript.

CONFLICTS OF INTEREST

All authors have completed the ICMJE uniform disclosure form at www.icmje.org/coi_disclosure.pdf and declare: NIHR CLAHRC West provided financial support for the submitted work; RM has received grants from Cancer Research UK in the previous three years; no other relationships or activities that could appear to have influenced the submitted work.

AUTHORS CONTRIBUTIONS

SM performed the literature review that informed this protocol. SM drafted the introduction, methods and dissemination sections. RM and MM drafted the statistical analysis outline. All authors review the draft manuscript, commented and amended it, and approved the final submission.

For peer review only

References

1. ONS. Cancer Registration Statistics, England: 2014. 2016 May 26;:1–17.

2. ONS. Cancer survival in England: Patients diagnosed between 2010 and 2014 and followed up to 2015. 2016 Sep pp. 1–15.

3. NICE. Suspected cancer: recognition and referral. 2015 Aug 26;:1–95.

4. Andriole GL, Crawford ED, Grubb RL III, Buys SS, Chia D, Church TR, et al. Mortality Results from a Randomized Prostate-Cancer Screening Trial. N Engl J Med. 2009 Mar 26;360(13):1310–9.

5. Schröder FH, Hugosson J, Roobol MJ, Tammela TLJ, Zappa M, Nelen V, et al. ArticlesScreening and prostate cancer mortality: results of theEuropean Randomised Study of Screening for ProstateCancer (ERSPC) at 13 years of follow-up. The Lancet. Elsevier Ltd; 2014 Aug 6;384(9959):1–9.

6. Jessen K, Søndergaard J, Larsen PV, Thomsen JL. Danish General Practitioners' Use of Prostate-Specific Antigen in Opportunistic Screening for Prostate Cancer: A Survey Comprising 174 GPs. International Journal of Family Medicine. 2013;2013(9):1–6.

7. Clements A, Watson E, Rai T, Bukach C, Shine B, Austoker J. The PSA testing dilemma: GPs' reports of consultations with asymptomatic men: a qualitative study. BMC Family Practice [Internet]. BioMed Central; 2007 Jun 25;8(1):35. Available from: http://www.biomedcentral.com/1471-2296/8/35

8. Cohen P, Frydenberg M, Borland R, Reading D, Clarke V, Hill D. Knowledge, attitudes and experience associated with testing for prostate cancer: a comparison between male doctors and men in the community. Internal Medicine Journal. 2002 Apr 17;32:215–23.

9. Drummond FJ, Carsin A-E, Sharp L, Comber H. Factors prompting PSA-testing of asymptomatic men in a country with no guidelines: a national survey of general practitioners. BMC Family Practice. 2009;10(1):3.

10. Evans R, Edwards AGK, Elwyn G, Watson E, Grol R, Brett J, et al. “It”s a maybe test’: men's experiences of prostate specific antigen testing in primary care. British Journal of General Practice. 2007 Apr 1;57:303–10.

11. Gronberg H, Adolfsson J, Aly M, NordstrÃm T, Wiklund P, Brandberg Y, et al. Prostate cancer screening in men aged 50-69 years (STHLM3): a prospective population-based diagnostic study. Lancet Oncology. Elsevier Ltd; 2015 Nov 24;16(16):1667–76.

12. Hamdy FC, Donovan JL, Lane JA, Mason M, Metcalfe C, Holding P, et al. 10-Year Outcomes after Monitoring, Surgery, or Radiotherapy for Localized Prostate Cancer. N Engl J Med. 2016 Sep 14;:NEJMoa1606220–10.

13. Donovan JL, Hamdy FC, Lane JA, Mason M, Metcalfe C, Walsh E, et al. Patient-Reported Outcomes after Monitoring, Surgery, or Radiotherapy for

For peer review only

Prostate Cancer. N Engl J Med. 2016 Sep 14;:NEJMoa1606221–14.

14. Bill-Axelson A, Holmberg L, Ruutu M, Garmo H, Stark JR, Busch C, et al. Radical Prostatectomy versus Watchful Waiting in Early Prostate Cancer. N Engl J Med. 2011 May 5;364(18):1708–17.

15. Resnick MJ, Koyama T, Fan K-H, Albertsen PC, Goodman M, Hamilton AS, et al. Long-Term Functional Outcomes after Treatment for Localized Prostate Cancer. N Engl J Med. 2013 Jan 31;368(5):436–45.

16. Sanda MG, Dunn RL, Michalski J, Sandler HM, Northouse L, Hembroff L, et al. Quality of Life and Satisfaction with Outcome among Prostate-Cancer Survivors. N Engl J Med. 2008 Mar 20;358(12):1250–61.

17. Smith DP, King MT, Egger S, Berry MP, Stricker PD, Cozzi P, et al. Quality of life three years after diagnosis of localised prostate cancer: population based cohort study. BMJ. 2009 Nov 27;339(nov27 2):b4817–7.

18. Wilt TJ, Brawer MK, Jones KM, Barry MJ, Aronson WJ, Fox S, et al. Radical Prostatectomy versus Observation for Localized Prostate Cancer. N Engl J Med. 2012 Jul 19;367(3):203–13.

19. Eisenhauer EA, Therasse P, Bogaerts J, Schwartz LH, Sargent D, Ford R, et al. New response evaluation criteria in solid tumours: Revised RECIST guideline (version 1.1). 2009 Jan 1;45(2):228–47. Available from: http://dx.doi.org/10.1016/j.ejca.2008.10.026

20. de Camargo Cancela M, Comber H, Sharp L. Age remains the major predictor of curative treatment non-receipt for localised prostate cancer: a population-based study. British Journal of Cancer. Nature Publishing Group; 2013 May 30;109(1):272–9.

21. Akbari MR, Wallis CJD, Toi A, Trachtenberg J, Sun P, Narod SA, et al. The impact of a BRCA2 mutation on mortality from screen-detected prostate cancer. British Journal of Cancer. Nature Publishing Group; 2014 Aug 7;111(6):1238–40.

22. Cantarutti A, Bonn SE, Adami H-O, Grönberg H, Bellocco R, Bälter K. Body mass index and mortality in men with prostate cancer. Prostate. 2015 Apr 30;75(11):1129–36.

23. Van Roermund JGH, Hinnen KA, Battermann JJ, Witjes JA, Bosch JLHR, Kiemeney LA, et al. Body mass index is not a prognostic marker for prostate-specific antigen failure and survival in Dutch men treated with brachytherapy. BJU Int. 2009 Dec 8;105(1):42–8.

24. Chamie K, Daskivich TJ, Kwan L, Labo J, Dash A, Greenfield S, et al. Comorbidities, Treatment and Ensuing Survival in Men with Prostate Cancer. J GEN INTERN MED. 2011 Sep 21;27(5):492–9.

25. Omalu BI, Hammers JL, Parwani AV, Balani J, Shakir A, Ness RB. Is there an association between coronary atherosclerosis and carcinoma of the prostate in

For peer review only

men aged 50 years and older? An autopsy and coroner based post-mortem study. Niger J Clin Pract. 2013;16(1):45–4.

26. Lee J, Giovannucci E, Jeon JY. Diabetes and mortality in patients with prostate cancer: a meta-analysis. SpringerPlus. Springer International Publishing; 2016 Sep 13;:1–11.

27. Ørsted DD, Bojesen SE, Nielsen SF, Nordestgaard BG. Association of Clinical Benign Prostate Hyperplasia with Prostate Cancer Incidence and Mortality Revisited: A Nationwide Cohort Study of 3 009 258 Men. European Urology. 2011 Oct;60(4):691–8.

28. Tewari R, Rajender S, Natu SM, Dalela D, Goel A, Goel MM, et al. Diet, Obesity, and Prostate Health: Are We Missing the Link? Journal of Andrology. 2012 Sep 19;33(5):763–76.

29. Vance TM, Azabdaftari G, Pop EA, Lee SG, Su LJ, Fontham ETH, et al. Intake of dietary antioxidants is inversely associated with biomarkers of oxidative stress among men with prostate cancer. Br J Nutr. 2015 Nov 2;115(01):68–74.

30. Goetzl MA, VanVeldhuizen PJ, Thrasher JB. Effects of soy phytoestrogens on the prostate. Prostate Cancer Prostatic Dis. 2007 Feb 20;10(3):216–23.

31. Ngo TC, Lee JJ, Brooks JD, Nolley R, Ferrari M, Presti JC. Smoking and adverse outcomes at radical prostatectomy. URO. Elsevier Inc; 2013 Aug 1;31(6):749–54.

32. Rohrmann S, Genkinger JM, Burke A, Helzlsouer KJ, Comstock GW, Alberg AJ, et al. Smoking and Risk of Fatal Prostate Cancer in a Prospective U.S. Study. Urology. 2007 Apr;69(4):721–5.

33. Huncharek M, Haddock S, Reid R, Kupelnick B. Smoking as a Risk Factor for Prostate Cancer: A Meta-Analysis of 24 Prospective Cohort Studies. 2010 Mar 4;:1–9.

34. Chavarro JE, Stampfer MJ, Li H, Campos H, Kurth T, Ma J. A Prospective Study of Polyunsaturated Fatty Acid Levels in Blood and Prostate Cancer Risk. Cancer Epidemiology Biomarkers & Prevention. 2007 Jul 1;16(7):1364–70.

35. Salgado-Montilla J, Salgado MS, Trautmann BS, Sánchez-Ortiz R, Irizarry-Ramírez M. Association of serum lipid levels and prostate cancer severity among Hispanic Puerto Rican men. Lipids in Health and Disease. Lipids in Health and Disease; 2015 Sep 15;:1–7.

36. Olivan M, Rigau M, Colás E, Garcia M, Montes M, Sequeiros T, et al. Simultaneous Treatment with Statins and Aspirin Reduces the Risk of Prostate Cancer Detection and Tumorigenic Properties in Prostate Cancer Cell Lines. BioMed Research International. 2015;2015(4):1–11.

37. Chan JM, Kenfield SA, Paciorek A, Platz EA, Giovannucci EL, Stampfer MJ. Postdiagnostic Statin Use and the Risk of Lethal Prostate Cancer in the Health

For peer review only

Professionals Follow-up Study. Cancer Epidemiology Biomarkers & Prevention. 2015 Sep 30;24(10):1638–40.

38. Assayag J, Pollak MN, Azoulay L. Post-diagnostic use of beta-blockers and the risk of death in patients with prostate cancer. European Journal of Cancer. Elsevier Ltd; 2014 Nov 1;50(16):2838–45.

39. Neslund-Dudas C, Kandegedara A, Kryvenko ON, Gupta N, Rogers C, Rybicki BA, et al. Prostate Tissue Metal Levels and Prostate Cancer Recurrence in Smokers. Biol Trace Elem Res. 2014 Jan 4;157(2):107–12.

40. Schiffmann J, Beyer B, Tennstedt P, Boehm K, Mehring G, Schlomm T, et al. Oncological outcome after radical prostatectomy: Marital status does not make a difference. Int J Urol. 2015 Mar 17;22(5):484–9.

41. Raval AD, Thakker D, Vyas A, Salkini M, Madhavan S, Sambamoorthi U. Impact of metformin on clinical outcomes among men with prostate cancer: a systematic review and meta-analysis. Prostate Cancer Prostatic Dis. 2015 Feb 10;18(2):110–21.

42. Ahn J, Peters U, Albanes D, Purdue MP, Abnet CC, Chatterjee N, et al. Serum Vitamin D Concentration and Prostate Cancer Risk: A Nested Case-Control Study. JNCI Journal of the National Cancer Institute. 2008 Jun 3;100(11):796–804.

43. Bai X-Y, Qu X, Jiang X, Xu Z, Yang Y, Su Q, et al. Association between Dietary Vitamin C Intake and Risk of Prostate Cancer: A Meta-analysis Involving 103,658 Subjects. J Cancer. 2015 Jul 26;6(9):913–21.

44. Shang Y, Han G, Li J, Zhao J, Cui D, Liu C, et al. Vasectomy and prostate cancer risk: a meta-analysis of cohort studies. Sci Rep. 2015 Apr 30;5:9920–5.

45. Majeed A, Car J, Sheikh A. Accuracy and completeness of electronic patient records in primary care. Family Practice. Oxford University Press; 2008 Aug;25(4):213–4.

46. CPRD. Welcome to the Clinical Practice Research Datalink [Internet]. [cited 2016 Sep 18]. Available from: https://www.cprd.com/home/

47. Hippisley-Cox J, Coupland C. Development and validation of risk prediction algorithms to estimate future risk of common cancers in men and women: prospective cohort study. BMJ Open [Internet]. British Medical Journal Publishing Group; 2015 Mar 17;5(3):e007825–6. Available from: http://dx.doi.org/10.1136/bmjopen-2015-007825

48. Azur MJ, Stuart EA, Frangakis C, Leaf PJ. Multiple imputation by chained equations: what is it and how does it work? Int J Methods Psychiatr Res. John Wiley & Sons, Ltd; 2011 Feb 24;20(1):40–9.

49. Royston P, Parmar MK. Flexible parametric proportional-hazards and proportional-odds models for censored survival data, with application to prognostic modelling and estimation of treatment effects. Statist Med. 2002 Jul

For peer review only

2;21:2175–97.

50. Royston P, Ambler G, Sauerbrei W. The use of fractional polynomials to model continuous risk variables in epidemiology. International Journal of Epidemiology. 1999 Nov 3;28:964–74.

51. Hinchliffe SR, Lambert PC. Flexible parametric modelling of cause-specific hazards to estimate cumulative incidence functions. BMC Med Res Methodol. BMC Medical Research Methodology; 2013 Feb 6;13(13):1–14.

For peer review only

BMJ Open

Predicting prostate cancer progression: protocol for a retrospective cohort study to identify prognostic factors for prostate cancer outcomes using routine primary care data.

Journal:	*BMJ Open*
Manuscript ID	bmjopen-2017-019409.R1
Article Type:	Protocol
Date Submitted by the Author:	27-Nov-2017
Complete List of Authors:	Merriel, Samuel; Univ Bristol, Centre for Academic Primary Care<br>Martin, Richard; University of Bristol, Population Health Sciences<br>May, Margaret; University of Bristol, Population Health Sciences
**Primary Subject Heading**:	Urology
Secondary Subject Heading:	Epidemiology, Oncology
Keywords:	Epidemiology < ONCOLOGY, Urological tumours < ONCOLOGY, Prostate disease < UROLOGY

SCHOLARONE™
Manuscripts

ew only

‘Predicting prostate cancer progression: protocol for a retrospective cohort study to identify prognostic factors for prostate cancer outcomes using routine primary care data.’

Dr Samuel W D Merriel[1], Prof Margaret T May[2], Prof Richard M Martin[2]

1 Centre for Academic Primary Care, University of Bristol, UK

2 Population Health Sciences, University of Bristol, UK

Corresponding author

Dr Samuel William David Merriel

Centre for Academic Primary Care

Bristol Medical School

University of Bristol

39 Whatley Road

Clifton, Bristol, UK

Sam.merriel@bristol.ac.uk

0117 928 9000

Keywords – prostate cancer, prognosis, primary care

Word count – 1566

For peer review only

Abstract

Introduction

Prostate cancer is the most common cancer in UK males, with nearly 40,000 men diagnosed in 2014, and the second commonest cause of male cancer-related mortality. The clinical conundrum is that most men live with prostate cancer rather than die from it, while existing treatments have significant associated morbidity. Recent studies have shown very low mortality rates (1% after a median 10-years follow-up), and no treatment related reductions in mortality, in men with localised prostate cancer. This study will identify prognostic factors associated with prostate cancer progression to help differentiate aggressive from more indolent tumours in men with localised disease at diagnosis, and so inform the decision to adopt conservative (active surveillance) or radical (surgery or radiotherapy) management strategies.

Methods & analysis

The Clinical Practice Research Datalink (CPRD) contains 57,318 men who were diagnosed with prostate cancer between 01/01/1987 and 31/12/2016. These men will be linked to the Office for National Statistics (ONS) and the National Cancer Registration and Analysis Service (NCRAS) registry databases for mortality, TNM stage, Gleason grade, and treatment data. Men with a diagnosis date prior to 01/01/1987 and men with lymph node or distant metastases at diagnosis will be excluded. A priori determined prognostic factors potentially associated with prostate cancer mortality, the end point of cancer progression, will be measured at baseline, and the participants followed through to development of cancer progression, death, or the end of the follow-up period (31/12/2016). Cox proportional hazards regression will be used to estimate crude and mutually adjusted hazard ratios. Mortality risk will be predicted using flexible parametric survival models that can accurately fit the shape of the hazard function.

Ethics & dissemination

This study protocol has approval from the Independent Scientific Advisory Committee (ISAC) for the UK Medicines and Healthcare products Regulatory Agency (MHRA) Database

For peer review only

Research (protocol 17_041). The findings will be presented in peer reviewed journals and local CPRD researcher meetings.

For peer review only

Strengths and limitations

- The study cohort is drawn from the CPRD, a large, representative UK primary care dataset, with linked cancer registry and ONS data
- Predicting cancer progression is a more clinically useful outcome than simply detecting localised disease in patients with a disease that is often indolent and slow-growing
- Flexible parametric modelling will be used to control for the effects of intermediate variables and prognostic factor combinations will be used
- Cancer registry data and ONS mortality data are not available for the full study period, and CPRD data may not be complete

For peer review only

Introduction

Prostate cancer is the most commonly diagnosed cancer in males, and the second most commonly diagnosed cancer in the United Kingdom (UK). 39,741 new cases of prostate cancer were diagnosed in England in 2014, with an age-standardised incidence rate of 177.8 per 100,000 males(1). In the same year there were approximately 11,300 prostate cancer deaths in the UK, making prostate cancer the second most common cause of cancer death in males. Nevertheless, survival rates for prostate cancer are relatively high compared to other cancer types. The overall 5-year age-standardised net survival for men with prostate cancer in England was 83.6%, and the predicted 10-year survival for men in England diagnosed with prostate cancer in 2015 is 79.9%(2). This suggests many men diagnosed with prostate cancer have indolent disease. A key clinical conundrum relates to distinguishing men with slow-growing tumours that could be managed conservatively with active monitoring from more aggressive, potentially fatal disease that may require more radical intervention.

Prostate cancer can be detected in men in different ways. GPs need to consider the possibility of prostate cancer in men presenting with lower urinary tract symptoms, erectile dysfunction or visible haematuria. Asymptomatic men may also be found to have raised prostate specific antigen (PSA) levels, and need to be referred for further investigation(3). However, the use of PSA as a screening and prognostic biomarker remains controversial(4,5), and GPs(6-9), and patients(10) have mixed views about its utility in informing investigation and treatment decisions for prostate cancer. Other screening methods for predicting prostate cancer severity have been tested, such as the STHLM-3 model(11), which did not look at risk of progression and relies on genetic biomarkers that are not readily available in primary care at this time.

The Bristol-based *ProtecT* multi-centre trial randomised men with clinically localised prostate cancer to either active monitoring, radical surgery (prostatectomy) or radical radiotherapy. After a median of 10 years' follow-up there was no difference in prostate cancer mortality. Overall, the 10-year mortality rates were very low (1%), and men randomised to active monitoring were at an increased risk of clinical progression and development of metastatic disease (22.9 per 1,000 person years follow-up) compared to the

For peer review only

radical treatment arms (8.9 and 9 per 1,000 person years respectively).(12) Men receiving surgery or radiotherapy reported more adverse effects on urinary, sexual and bowel function compared to the active monitoring cohort.(13) This was broadly consistent with other studies of prostate cancer treatment.(14-18) Identifying factors associated with prostate cancer progression may help determine the risk for men having more aggressive prostate cancer, and inform shared decision making about whether to undergo radical treatments or choose active monitoring.

Cancer progression is well defined is cancer treatment trials, following the widely used RECIST criteria.(19) However, the concept of cancer progression in prognostic studies is much less well defined or consistently applied. The *ProtecT* trial defined prostate cancer progression as the occurrence of any of the following events: evidence of metastasis, development of T3/T4 disease, commencing long-term androgen therapy, ureteric obstruction, rectal fistula, and new need for catheter.(12) Several prognostic factors have been identified that may be associated with prostate cancer mortality, the endpoint of prostate cancer progression. These include demographic(20), genetic(21), physiological(22,23), co-morbidity(24-27), lifestyle(28-33), biochemical(34,35) and medication(36,37) factors. The strength of evidence for these prognostics factors varies, and for many others it is conflicting(38-44).

Primary care medical records contain a wealth of information on a patient's medical history, medications, family history, and investigation results(45). The Clinical Practice Research Datalink (CPRD)(46) is a large UK primary care research database representative of the general population, with links to many other relevant healthcare data registries and Office for National Statistics (ONS) data. This information is already used for many risk prediction tools in primary care settings to predict outcomes and inform treatment decisions. Examples include QCancer(47), which predicts a patient's absolute risk of future cancer diagnosis. To date there are no risk prediction tools for cancer progression used in clinical practice.

This study aims to establish which risk factors are associated with prostate cancer progression using primary care medical records data. These findings, in combination with

For peer review only

metabolomic and genomic data, will inform the development of a clinical risk prediction model for the progression of prostate cancer following diagnosis.

For peer review only

Methods and analysis

Within the CPRD dataset, at least 57,318 men had a diagnosis of prostate cancer made between 01/01/1987 and 31/12/2016, of whom 22,080 have a recorded date of death. These men will form the basis of the study cohort. Additional mortality, staging (TNM and gleason grade), and treatment data will be obtained by using each man's NHS number to link them to the ONS (available from 01/01/1998) and National Cancer Registration and Analysis Service (NCRAS) (available from 01/01/1990) databases. The index date will be the date the diagnosis of prostate cancer was first entered into the primary care medical record. From this date, the men will be followed until the date of their death, the development of prostate cancer progression, or the end of the cohort period, whichever is later. Men with a diagnosis date prior to 01/01/1987, and men with lymph node or distant metastases at diagnosis, will be excluded from the analysis.

Each of the hypothesised prognostic factors for prostate cancer mortality identified a priori (see Table 1) will be recorded as an 'exposure' if it is entered into the patient record at the study baseline (index date), or recorded before the diagnosis of prostate cancer is entered. Continuous variables, such as height, weight and the biochemical markers, will be measured according to the most recent result prior to the coding of a diagnosis of prostate cancer within the study time period. Genetic factors, lifestyle exposures, medications and co-morbidities will be considered in a binary manner in relation to their presence or absence at the index date. Missing data will be controlled for using multiple imputation methods.(48)

**Category**	**Prognostic factor(s)**	**Definition/unit**
Basic demographics	Age, date of birth	Years
	Post code	Current patient address
	Ethnicity	ONS ethnicity categories
Physiological	Height	Centimetres (cm)
	Weight	Kilograms (kg)
	Waist circumference	Centimetres (cm)
	Waist:hip ratio	

For peer review only

Genetic	Family history of prostate cancer	Recorded diagnosis in first- or second-degree relative
Biochemical	Triglycerides, HDL cholesterol, LDL cholesterol, VLDL cholesterol	mmol/L
	HbA1c	mmol/L
	CRP	mg/L
	Ferritin	ug/L
	Haemoglobin	g/L
	Albumin	g/L
	Serum glucose, plasma glucose	mmol/L
	Lead	ug/L
Lifestyle	Smoking history	Smoking tobacco prior to or at index date
	Relationship status	Patient identifies as being in a relationship
	Alcohol intake	Units per week
Medications	Simvastatin, Atorvastatin, Metformin, Aspirin, Atenolol, Bisoprolol, Sotalol, Labetalol, Carvedilol, Nebivolol, Metoprolol, Propanolol, Finasteride, Dutasteride, Colecalciferol, Ergocalciferol, Alfacalcidol	Prescribed within the 12 months prior to index date
Co-morbidities	Type 2 Diabetes Mellitus, Ischaemic heart disease, Stroke, Peripheral vascular disease, Benign prostatic hypertrophy, Chronic Obstructive Pulmonary Disease (COPD)	Diagnosed prior to index date

Table 1 – Prognostic factors to be assessed

HDL – High Density Lipoprotein; LDL – Low Density Lipoprotein; VLDL – Very Low Density Lipoprotein; HbA1c – Haemoglobin A1c; CRP – C-Reactive Protein

For peer review only

To achieve 95% power and detect a difference in hazard ratios of 0.5 in prostate cancer mortality for a binary risk factor using an alpha of 0.05, a sample of at least 8,762 men with prostate cancer would be required, assuming a 1% annual mortality rate over a median 10-year follow-up.

The primary outcome measure will be prostate cancer mortality. Participants will be presumed to be alive at the end of the follow-up period if they have not been reported as deceased according to the ONS mortality data. Secondary outcome measures of prostate cancer progression will include all-cause mortality, change from localised to metastatic disease, and commencing anti-androgen therapy or chemotherapy. We will use whether the treatment recorded in the registry is stated to be localised (i.e. 1 tumour treated) or systemic (i.e. >1 tumour treated) to help distinguish between early and advanced disease.

Descriptive statistics will be used to summarise the basic demographic details of the men. The prevalence of the pre-selected putative prognostic factors will be calculated and presented. Cox proportional hazards regression will be used to estimate the crude and mutually adjusted hazard ratios (with 95% confidence intervals) for prostate cancer and all-cause mortality according to the prognostic factors. Related prognostic factors, such as smoking and COPD, will also be grouped to account for potential intermediate variables. This analysis will be repeated with stratification by stage at diagnosis. In order to allow for flexibility in the shape of the cumulative hazard function we will use flexible parametric survival models(49) for prognostic modelling. These models incorporate cubic spline terms in the log cumulative hazard function and are based on weibull, loglogistic or lognormal distributions of survival time. We will check for non-linearities in the effects of continuous predictors using fractional polynomials(50) and also test for time varying effects of prognostic factors. We will determine mortality risk in groups defined by important prognostic factors. To asses competing risks, we will use cause–specific survival analysis to estimate at 1, 2, 5 and 10 years post prostate cancer diagnosis the contribution of prostate cancer mortality to overall mortality in those who have died by prognostic factor combinations(51).

For peer review only

Ethics and dissemination

This study protocol has approval from the Independent Scientific Advisory Committee (ISAC) for the UK Medicines and Healthcare products Regulatory Agency (MHRA) Database Research (protocol 17_041).

The findings of this study will be submitted as a manuscript to peer reviewed journal to aid dissemination to clinicians and other researchers in the field. It will also be presented and discussed at local CPRD working groups to inform other researchers' methods using the CPRD database. Subsequent studies of the prediction tool, based on this piece of research, will involve clinicians at every stage to ensure the final tool is acceptable for use in clinical practice.

For peer review only

Conclusions

This study will lay the foundation for the development of a clinically useful risk prediction tool. Clinicians will be able to use the tool, inputting routine primary care data, to improve shared decision making about an individual's prognosis and, if validated and shown effective in trials, inform their practice when deciding with patients whether to undergo radical surgery or radiotherapy or be followed up conservatively using active monitoring.

Patients will also benefit from this work in other ways. They will be able to receive more information from GPs and NHS specialists about the risk of progression of their prostate cancer, and they will be able to decide within a shared decision-making framework with their doctors about the potential benefits and harms of undergoing radical treatment or active monitoring.

For peer review only

FUNDING

This project is in part funded by the NIHR CLAHRC West. SM is the recipient of an NIHR academic clinical fellowship.

DATA SHARING STATEMENT

There is no data to share from this protocol manuscript.

CONFLICTS OF INTEREST

All authors have completed the ICMJE uniform disclosure form at www.icmje.org/coi_disclosure.pdf and declare: NIHR CLAHRC West provided financial support for the submitted work; RM has received grants from Cancer Research UK in the previous three years; no other relationships or activities that could appear to have influenced the submitted work.

The views expressed are those of the authors and not necessarily those of the NHS, the NIHR, or the UK Department of Health.

AUTHORS CONTRIBUTIONS

SM performed the literature review that informed this protocol. SM drafted the introduction, methods and dissemination sections. RM and MM drafted the statistical analysis outline. All authors review the draft manuscript, commented and amended it, and approved the final submission.

For peer review only

References

1. ONS. Cancer Registration Statistics, England: 2014. 2016 May 26;:1–17.

2. ONS. Cancer survival in England: Patients diagnosed between 2010 and 2014 and followed up to 2015. 2016 Sep pp. 1–15.

3. NICE. Suspected cancer: recognition and referral. 2015 Aug 26;:1–95.

4. Andriole GL, Crawford ED, Grubb RL III, Buys SS, Chia D, Church TR, et al. Mortality Results from a Randomized Prostate-Cancer Screening Trial. N Engl J Med. 2009 Mar 26;360(13):1310–9.

5. Schröder FH, Hugosson J, Roobol MJ, Tammela TLJ, Zappa M, Nelen V, et al. ArticlesScreening and prostate cancer mortality: results of theEuropean Randomised Study of Screening for ProstateCancer (ERSPC) at 13 years of follow-up. The Lancet. Elsevier Ltd; 2014 Aug 6;384(9959):1–9.

6. Jessen K, Søndergaard J, Larsen PV, Thomsen JL. Danish General Practitioners' Use of Prostate-Specific Antigen in Opportunistic Screening for Prostate Cancer: A Survey Comprising 174 GPs. International Journal of Family Medicine. 2013;2013(9):1–6.

7. Clements A, Watson E, Rai T, Bukach C, Shine B, Austoker J. The PSA testing dilemma: GPs' reports of consultations with asymptomatic men: a qualitative study. BMC Family Practice [Internet]. BioMed Central; 2007 Jun 25;8(1):35. Available from: http://www.biomedcentral.com/1471-2296/8/35

8. Cohen P, Frydenberg M, Borland R, Reading D, Clarke V, Hill D. Knowledge, attitudes and experience associated with testing for prostate cancer: a comparison between male doctors and men in the community. Internal Medicine Journal. 2002 Apr 17;32:215–23.

9. Drummond FJ, Carsin A-E, Sharp L, Comber H. Factors prompting PSA-testing of asymptomatic men in a country with no guidelines: a national survey of general practitioners. BMC Family Practice. 2009;10(1):3.

10. Evans R, Edwards AGK, Elwyn G, Watson E, Grol R, Brett J, et al. "It"s a maybe test': men's experiences of prostate specific antigen testing in primary care. British Journal of General Practice. 2007 Apr 1;57:303–10.

11. Gronberg H, Adolfsson J, Aly M, NordstrÃm T, Wiklund P, Brandberg Y, et al. Prostate cancer screening in men aged 50-69 years (STHLM3): a prospective population-based diagnostic study. Lancet Oncology. Elsevier Ltd; 2015 Nov 24;16(16):1667–76.

12. Hamdy FC, Donovan JL, Lane JA, Mason M, Metcalfe C, Holding P, et al. 10-Year Outcomes after Monitoring, Surgery, or Radiotherapy for Localized Prostate Cancer. N Engl J Med. 2016 Sep 14;:NEJMoa1606220–10.

13. Donovan JL, Hamdy FC, Lane JA, Mason M, Metcalfe C, Walsh E, et al. Patient-Reported Outcomes after Monitoring, Surgery, or Radiotherapy for

For peer review only - http://bmjopen.bmj.com/site/about/guidelines.xhtml

Prostate Cancer. N Engl J Med. 2016 Sep 14;:NEJMoa1606221–14.

14. Bill-Axelson A, Holmberg L, Ruutu M, Garmo H, Stark JR, Busch C, et al. Radical Prostatectomy versus Watchful Waiting in Early Prostate Cancer. N Engl J Med. 2011 May 5;364(18):1708–17.

15. Resnick MJ, Koyama T, Fan K-H, Albertsen PC, Goodman M, Hamilton AS, et al. Long-Term Functional Outcomes after Treatment for Localized Prostate Cancer. N Engl J Med. 2013 Jan 31;368(5):436–45.

16. Sanda MG, Dunn RL, Michalski J, Sandler HM, Northouse L, Hembroff L, et al. Quality of Life and Satisfaction with Outcome among Prostate-Cancer Survivors. N Engl J Med. 2008 Mar 20;358(12):1250–61.

17. Smith DP, King MT, Egger S, Berry MP, Stricker PD, Cozzi P, et al. Quality of life three years after diagnosis of localised prostate cancer: population based cohort study. BMJ. 2009 Nov 27;339(nov27 2):b4817–7.

18. Wilt TJ, Brawer MK, Jones KM, Barry MJ, Aronson WJ, Fox S, et al. Radical Prostatectomy versus Observation for Localized Prostate Cancer. N Engl J Med. 2012 Jul 19;367(3):203–13.

19. Eisenhauer EA, Therasse P, Bogaerts J, Schwartz LH, Sargent D, Ford R, et al. New response evaluation criteria in solid tumours: Revised RECIST guideline (version 1.1). 2009 Jan 1;45(2):228–47. Available from: http://dx.doi.org/10.1016/j.ejca.2008.10.026

20. de Camargo Cancela M, Comber H, Sharp L. Age remains the major predictor of curative treatment non-receipt for localised prostate cancer: a population-based study. British Journal of Cancer. Nature Publishing Group; 2013 May 30;109(1):272–9.

21. Akbari MR, Wallis CJD, Toi A, Trachtenberg J, Sun P, Narod SA, et al. The impact of a BRCA2 mutation on mortality from screen-detected prostate cancer. British Journal of Cancer. Nature Publishing Group; 2014 Aug 7;111(6):1238–40.

22. Cantarutti A, Bonn SE, Adami H-O, Grönberg H, Bellocco R, Bälter K. Body mass index and mortality in men with prostate cancer. Prostate. 2015 Apr 30;75(11):1129–36.

23. Van Roermund JGH, Hinnen KA, Battermann JJ, Witjes JA, Bosch JLHR, Kiemeney LA, et al. Body mass index is not a prognostic marker for prostate-specific antigen failure and survival in Dutch men treated with brachytherapy. BJU Int. 2009 Dec 8;105(1):42–8.

24. Chamie K, Daskivich TJ, Kwan L, Labo J, Dash A, Greenfield S, et al. Comorbidities, Treatment and Ensuing Survival in Men with Prostate Cancer. J Gen Intern Med. 2011 Sep 21;27(5):492–9.

25. Omalu BI, Hammers JL, Parwani AV, Balani J, Shakir A, Ness RB. Is there an association between coronary atherosclerosis and carcinoma of the prostate in

For peer review only

men aged 50 years and older? An autopsy and coroner based post-mortem study. Niger J Clin Pract. 2013;16(1):45–4.

26. Lee J, Giovannucci E, Jeon JY. Diabetes and mortality in patients with prostate cancer: a meta-analysis. SpringerPlus. Springer International Publishing; 2016 Sep 13;:1–11.

27. Ørsted DD, Bojesen SE, Nielsen SF, Nordestgaard BG. Association of Clinical Benign Prostate Hyperplasia with Prostate Cancer Incidence and Mortality Revisited: A Nationwide Cohort Study of 3 009 258 Men. European Urology. 2011 Oct;60(4):691–8.

28. Tewari R, Rajender S, Natu SM, Dalela D, Goel A, Goel MM, et al. Diet, Obesity, and Prostate Health: Are We Missing the Link? Journal of Andrology. 2012 Sep 19;33(5):763–76.

29. Vance TM, Azabdaftari G, Pop EA, Lee SG, Su LJ, Fontham ETH, et al. Intake of dietary antioxidants is inversely associated with biomarkers of oxidative stress among men with prostate cancer. Br J Nutr. 2015 Nov 2;115(01):68–74.

30. Goetzl MA, VanVeldhuizen PJ, Thrasher JB. Effects of soy phytoestrogens on the prostate. Prostate Cancer Prostatic Dis. 2007 Feb 20;10(3):216–23.

31. Ngo TC, Lee JJ, Brooks JD, Nolley R, Ferrari M, Presti JC. Smoking and adverse outcomes at radical prostatectomy. URO. Elsevier Inc; 2013 Aug 1;31(6):749–54.

32. Rohrmann S, Genkinger JM, Burke A, Helzlsouer KJ, Comstock GW, Alberg AJ, et al. Smoking and Risk of Fatal Prostate Cancer in a Prospective U.S. Study. Urology. 2007 Apr;69(4):721–5.

33. Huncharek M, Haddock S, Reid R, Kupelnick B. Smoking as a Risk Factor for Prostate Cancer: A Meta-Analysis of 24 Prospective Cohort Studies. 2010 Mar 4;:1–9.

34. Chavarro JE, Stampfer MJ, Li H, Campos H, Kurth T, Ma J. A Prospective Study of Polyunsaturated Fatty Acid Levels in Blood and Prostate Cancer Risk. Cancer Epidemiology Biomarkers & Prevention. 2007 Jul 1;16(7):1364–70.

35. Salgado-Montilla J, Salgado MS, Trautmann BS, Sánchez-Ortiz R, Irizarry-Ramírez M. Association of serum lipid levels and prostate cancer severity among Hispanic Puerto Rican men. Lipids in Health and Disease. Lipids in Health and Disease; 2015 Sep 15;:1–7.

36. Olivan M, Rigau M, Colás E, Garcia M, Montes M, Sequeiros T, et al. Simultaneous Treatment with Statins and Aspirin Reduces the Risk of Prostate Cancer Detection and Tumorigenic Properties in Prostate Cancer Cell Lines. BioMed Research International. 2015;2015(4):1–11.

37. Chan JM, Kenfield SA, Paciorek A, Platz EA, Giovannucci EL, Stampfer MJ. Postdiagnostic Statin Use and the Risk of Lethal Prostate Cancer in the Health

For peer review only

Professionals Follow-up Study. Cancer Epidemiology Biomarkers & Prevention. 2015 Sep 30;24(10):1638–40.

38. Assayag J, Pollak MN, Azoulay L. Post-diagnostic use of beta-blockers and the risk of death in patients with prostate cancer. European Journal of Cancer. Elsevier Ltd; 2014 Nov 1;50(16):2838–45.

39. Neslund-Dudas C, Kandegedara A, Kryvenko ON, Gupta N, Rogers C, Rybicki BA, et al. Prostate Tissue Metal Levels and Prostate Cancer Recurrence in Smokers. Biol Trace Elem Res. 2014 Jan 4;157(2):107–12.

40. Schiffmann J, Beyer B, Tennstedt P, Boehm K, Mehring G, Schlomm T, et al. Oncological outcome after radical prostatectomy: Marital status does not make a difference. Int J Urol. 2015 Mar 17;22(5):484–9.

41. Raval AD, Thakker D, Vyas A, Salkini M, Madhavan S, Sambamoorthi U. Impact of metformin on clinical outcomes among men with prostate cancer: a systematic review and meta-analysis. Prostate Cancer Prostatic Dis. 2015 Feb 10;18(2):110–21.

42. Ahn J, Peters U, Albanes D, Purdue MP, Abnet CC, Chatterjee N, et al. Serum Vitamin D Concentration and Prostate Cancer Risk: A Nested Case-Control Study. JNCI Journal of the National Cancer Institute. 2008 Jun 3;100(11):796–804.

43. Bai X-Y, Qu X, Jiang X, Xu Z, Yang Y, Su Q, et al. Association between Dietary Vitamin C Intake and Risk of Prostate Cancer: A Meta-analysis Involving 103,658 Subjects. J Cancer. 2015 Jul 26;6(9):913–21.

44. Shang Y, Han G, Li J, Zhao J, Cui D, Liu C, et al. Vasectomy and prostate cancer risk: a meta-analysis of cohort studies. Sci Rep. 2015 Apr 30;5:9920–5.

45. Majeed A, Car J, Sheikh A. Accuracy and completeness of electronic patient records in primary care. Family Practice. Oxford University Press; 2008 Aug;25(4):213–4.

46. CPRD. Welcome to the Clinical Practice Research Datalink [Internet]. [cited 2016 Sep 18]. Available from: https://www.cprd.com/home/

47. Hippisley-Cox J, Coupland C. Development and validation of risk prediction algorithms to estimate future risk of common cancers in men and women: prospective cohort study. BMJ Open [Internet]. British Medical Journal Publishing Group; 2015 Mar 17;5(3):e007825–6. Available from: http://dx.doi.org/10.1136/bmjopen-2015-007825

48. Azur MJ, Stuart EA, Frangakis C, Leaf PJ. Multiple imputation by chained equations: what is it and how does it work? Int J Methods Psychiatr Res. John Wiley & Sons, Ltd; 2011 Feb 24;20(1):40–9.

49. Royston P, Parmar MK. Flexible parametric proportional-hazards and proportional-odds models for censored survival data, with application to prognostic modelling and estimation of treatment effects. Statist Med. 2002 Jul

For peer review only

2;21:2175–97.

50. Royston P, Ambler G, Sauerbrei W. The use of fractional polynomials to model continuous risk variables in epidemiology. International Journal of Epidemiology. 1999 Nov 3;28:964–74.

51. Hinchliffe SR, Lambert PC. Flexible parametric modelling of cause-specific hazards to estimate cumulative incidence functions. BMC Med Res Methodol. BMC Medical Research Methodology; 2013 Feb 6;13(13):1–14.

For peer review only
